# Pollen-derived microcapsules for aspirin microencapsulation: *in vitro* release and physico-chemical studies†

**DOI:** 10.1039/d2ra02888c

**Published:** 2022-08-10

**Authors:** Al-Shymaa Y. Mohammed, Amro K. F. Dyab, Fouad Taha, Ahmed I. A. Abd El-Mageed

**Affiliations:** Colloids & Advanced Materials Group, Chemistry Department, Faculty of Science, Minia University Minia 61519 Egypt ahmed.abdelmageed@mu.edu.eg; Chemistry Department, Faculty of Science, GALALA University Galala City Suez 43711 Egypt

## Abstract

Aspirin, also known as acetylsalicylic acid (ASA), is one of the most crucial therapies needed and/or used in a basic health system. Using biocompatible materials to encapsulate ASA would improve its therapeutic efficacy and reduce its side effects *via* controlled release in physiological environments. Consequently, we explore in this study the feasibility of encapsulation of ASA into robust *Lycopodium clavatum* L. sporopollenin (LCS) microcapsules. After extracting sporopollenin from their natural micrometer-sized raw spores, the physico-chemical features of the extracted sporopollenin, pure ASA, and sporopollenin loaded with ASA were characterised using various methods, including optical microscopy, Fourier transform infrared spectroscopy (FTIR), ultraviolet-visible (UV-vis.) spectroscopy, thermogravimetric analysis (TGA), scanning electron microscopy (SEM), and X-ray diffraction (XRD). Additionally, we demonstrate the *in vitro* release profile of ASA in a triggered gastrointestinal environment utilizing kinetics analysis to investigate the mechanism of release. The LCS microcapsules were found to be excellent encapsulants for the crucial ASA drug and achieved controlled *in vitro* release, that would enable further investigations to rationally design versatile controlled delivery platforms.

## Abstract 2

Encapsulation of aspirin (ASA) inside natural sporopollenin microcapsules and *in vitro* controlled release under different gastrointestinal (GI) conditions.

## Graphical abstract

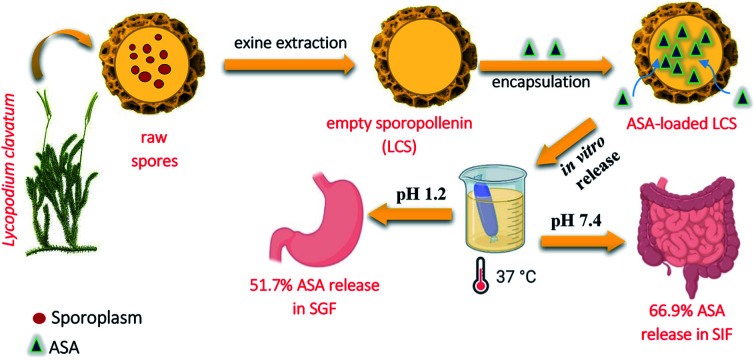

## Introduction

**Scheme 1:**
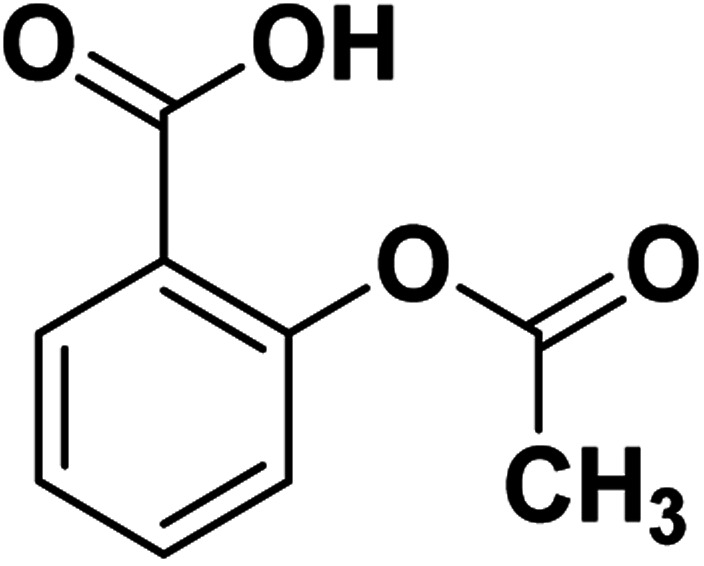
Structure of ASA.

Aspirin (acetylsalicylic acid, ASA) is one of the oldest well-known synthetic drugs.^1^ It was first synthesized in Germany and has remained one of the most common “over the counter” drugs of all time.^2^ ASA is one of the most often prescribed medicines worldwide.^3^ It is listed on the WHO's Model List of Essential Medicines, identifying the most critical pharmaceuticals necessary to an entire health system.^3^ The structure of acetylsalicylic acid or 2-acetoxybenzoic acid is presented in Scheme 1.

ASA is a phenomenal drug that can be used for a variety of conditions.^4,5^ Aspirin is a salicylate drug that is used therapeutically as an analgesic to relieve pains and aches (general pain reliever), as an antipyretic to reduce fever (fever depressant), as an anti-rheumatic, anticoagulant, platelet aggregation inhibitor, and as an anti-inflammatory drug.^6,7^

It is suggested to avoid both primary and secondary diabetes mellitus,^8^ and considered to be one of the most efficient antiplatelet agents for preventing cardiovascular death and myocardial infarction. Compared to numerous antiplatelet agents, ASA is the oldest, cheapest, and the most established antiplatelet agent.^9^ Aspirin is used for long-term medication; some people take aspirin every day with low dosages to reduce their risk of heart attacks and prevent strokes.^6^ It is also utilized to delay the onset of Alzheimer's disease.^10^ According to new evidence, ASA may also be an effective cancer-prevention tool.^6,11^

In spite of the prevalent use of ASA in treatment of several diseases, it has some limitations such as its short biological half-life and low solubility, so its pharmacological profile is poor.^12,13^ Also, aspirin irritates the stomach lining, leading to stomach ulcers in high dosages.^2^ Consequently, an appropriate dose must be used to minimize the adverse reaction of the gastrointestinal tract to aspirin. In pharmaceutical engineering, to avoid stomach injury, ASA is frequently synthesized as an enteric tablet or inclusion complex and then combined with other adjuvants.^14^ These mentioned challenges makes the development of effective controlled release formulations of ASA highly desirable to reduce dosing frequency and improve patient compliance.

Since ASA is insoluble (according to Biopharmaceutics Classification System) which lead to decrease the drug bio-availability. Hence, it's recommended to enhance the drug solubility through other techniques *i.e.* loading the drug on metallic nanoparticles, encapsulating in emulsions and/or natural microcapsules.^15,16^ This study also presents a solution for the ASA poor solubility.

Drug repurposing (applying known drugs *i.e.* ASA to new indications) has attracted significant attention because of its significant advantages over traditional approaches in terms of development time, cost, and safety that make it possible for researchers to measure knowledge evolution and the transfer of drug research.^17–19^ ASA could also significantly promote bone repair in rodents as reported for applications in bone tissue regeneration.^20,21^

Utilizing substrates to encapsulate medications, allowing them to release at a predetermined rate in a physiological environment, is referred to as controlled release.^2^ This drug delivery approach can ensure that the drug reaches the target at the right time, in the right place, and in an adequate amount, enhancing the localized therapeutic impact while minimizing side effects on other tissues and organs.^2^ The controlled release system can be formulated in a variety of forms, including microcapsules, microparticles, wafers, films, and implants.^22–26^

Microcapsules derived from plant pollens and spores have recently been proved to be a promising natural resource that enables a diverse range of robust microencapsulation applications.^27–29^ Spores from non-flowering plants and pollen grains from flowering plants acted as a protector for genetic components that were vulnerable to harsh environmental conditions.^30^

Typically, spores and pollen associated with plants have an outer shell termed the exine, an inner shell termed the intine, and cytoplasmic elements on the interior.^28^ The male gamete is encased in the intine, which is guarded by the exine, along with other macromolecules and nutrients. The intine is predominantly constituted of carbohydrates, including pectin and cellulose, whereas the exine is primarily composed of sporopollenin, a biopolymer.^31–34^ Sporopollenin was specified as “One of the most exceptional resisting materials known in the organic world”^35^ due to its resistance to many variables (*e.g*., acids, bases, high temperatures, and pressures).^35,36^ Sporopollenin can be utilized in many potential applications, including ion and ligand exchange, cosmetics,^37,38^ chemical micro-reactors, catalytic support, and solid-phase synthesis.^39,40^ It can be attributed to their magnificent properties such as chemical inertness, unique surface architecture, nano-porous channels, physical robustness, uniform size, large internal cavity, natural abundance, and their ability to block UV radiation effectively.^29,41^ Moreover, *Lycopodium clavatum* spores' some species have shown great efficiency in encapsulating a variety of encapsulates, including live cells,^42^ taste masking oils,^43,44^ proteins,^45^ inorganic nanoparticles,^39^ as well as drugs while retaining their herbal therapeutic advantages.^29,32,46–48^. Accordingly, these natural microcapsules might be compacted and used as tablets^29,49^ with or without enteric coating^50^ for safely administering patients.

Microencapsulation provides desirable properties such as targeting, drug stability, and controlled release, becoming increasingly important. In general, traditional microencapsulation techniques are expensive and problematic, especially regarding manufacturing monodisperse, uniform, and biodegradable microcapsules.

Many different approaches were used to isolate and/or functionalize sporopollenin microcapsules from pollen grains *i.e.* using ionic liquids as a green and simple chemical approach that enhance the removal of the intine-rich cell wall as well as the functional groups distribution on the exine surface.^51^ Other environmentally friendly chemical approach was reported using Fe-modified sporopollenin based on a hydrophobic ionic liquid to purify and functionalize sporopollenin (SP) with Fe-ions.^52^

Sporopollenin separated from natural spores outperformed synthetic microcapsules due to their distinct structural and morphological features.^29^ Natural sporopollenin's distinctive properties, most notably their size homogeneity, well-defined surface ornamentation, and the elasticity of their shells,^29^ make them ideal natural microencapsulant compared to synthetic microcapsules. An important feature of Sporopollenin is the biodegradation, as an indication for its potential in biomedical application. *Lycopodium clavatum* spores were shown to degrade in blood,^53,54^ whilst they appeared to remain stable in simulated gastric fluid (SGF) and simulated intestinal fluid (SIF).^55^

The current study focuses on the feasibility of encapsulating ASA into *Lycopodium clavatum* L. sporopollenin (LCS) to examine its delivery under distinct settings, thereby increasing its pharmacokinetic features and bioavailability. Moreover, we demonstrate the *in vitro* release profile of ASA in an activated gastrointestinal environment utilizing kinetics analysis to study the mechanism of release. Additionally, we indicate that LCS microcapsules are an excellent carrier for ASA encapsulation. To the best of our knowledge, no previous research has been published on the encapsulation and *in vitro* release of ASA using the cost-effective LCS microcapsules. The extracted sporopollenin, pure ASA, and sporopollenin loaded with ASA were also characterised physico-chemically utilizing various techniques (*e.g.*, SEM, Optical Microscopy, FTIR, UV-vis. Spectroscopy, XRD, and TGA).

## Materials and methods

### Materials

Raw *Lycopodium clavatum* L. (common club moss) spores (S-type, 28 μm) were obtained from Sigma-Aldrich, UK (Fig. S1†). Acetylsalicylic acid (ASA) (100%, molecular weight 180.159 g mol^−1^) was purchased from Sigma-Aldrich, UK. SERVA Electrophoresis GmbH dialysis membrane (SERVAPOR® dialysis tubing, MW. Cut-off 12–14 kDa), 16 mm diameter, regenerated cellulose, RC, packed dry (Germany), and standard white clips (closed length 45 mm) are available from Carl Roth GmbH + Co. KG Schoemperlenstraβe 3-5 D-76185 Karlsruhe, Germany. Other chemicals and characterization techniques (*e.g*., SEM, Optical Microscopy, Elemental Analysis, FTIR, UV-vis. Spectroscopy, XRD, TGA, and pH-meter) were mentioned in details in the ESI.†

### Methods

#### Extraction of sporopollenin microcapsules (LCS) from S-type *Lycopodium clavatum* L. spores

*Lycopodium clavatum* spores (50 g loose powder) were suspended in 375 mL acetone and refluxed for 4 hours with stirring. The defatted spores were filtered and left to dry in the open air overnight. They were then immersed in 375 mL aqueous potassium hydroxide solution at a concentration of 6% (w/v) and refluxed with stirring for 12 hours, followed by filtration and solution renewal after 6 hours. The mixture was then filtered through 20–25 μm Whatman filter paper, rinsed with hot water and hot ethanol, and dried overnight in the open air. Finally, the solid residue was immersed in 375 mL 85% *ortho*-phosphoric acid and refluxed for seven days with stirring. The suspension was then filtered again and washed with water, acetone, 2 M hydrochloric acid, 2 M sodium hydroxide, water, acetone, and finally with ethanol until a pure supernatant was gathered. It was then dried at 60 °C until a constant weight was reached. The sporopollenin produced was then kept at room temperature until future usage.

#### Encapsulation of aspirin into sporopollenin microcapsules LCS

In this work, we adopted a method that involved passive diffusion accompanied by vacuum loading. In a 5 mL glass tube, 200 mg of aspirin crystal was dissolved in 2 mL of ethanol and agitated for 30 minutes to ensure total dissolution. The aspirin solution was then passively loaded with 200 mg of dry LCS for 30 minutes stirring at 500 rpm (yielding 1 : 1 and/or 50% w/w drug loading, respectively). After passive loading, the glass tube was placed in a vacuum desiccator for an additional two hours for vacuum loading. The 1 : 1 ratio is called theoretical drug loading (TDL) and known well in pharmaceutical science. What we measure actually called loading capacity or drug loading (LC) or (DL). So, when we calculate the loading efficiency we use the following equation:Encapsulation efficiency (%) = (practical drug loading/theoretical drug loading) × 100

We have chosen this loading methods as well as this loading ratio as they produce the highest loading capacity (LC%) and encapsulation efficiency (EE%) values. Filtration was used to obtain the aspirin-loaded LCS microcapsules, which were then rinsed with 8 mL water and ethanol (1 : 1, v/v) to eliminate any surface-adhered medication. Finally, the ASA-loaded LCS were dried to a consistent weight at 78–79 °C and kept at room temperature until next usage.

#### *In vitro* ASA release from ASA-loaded microcapsules

We conducted ASA release studies from sporopollenin microcapsules in a basic medium enriched with 0.1 M NaOH, an acidic medium with a pH of 1.2 ± 0.05 (0.1 M HCl), and a neutral medium with a pH of 7.4 ± 0.02 (phosphate buffer saline, PBS). A pH of 1.2 was associated with simulated gastric fluid (SGF), whereas a pH of 7.4 was associated with simulated intestinal fluid (SIF), which mimicked gastrointestinal circumstances. The *in vitro* release experiments were carried out with a dialysis setup comprised of a dialysis bag and a receptor chamber (Fig. S2†). Ten milligrams of LCS microcapsules preloaded with ASA were considered carefully and dispersed in five milliliters of each release medium. The microcapsules were then transferred to a dialysis membrane (MW. Cut-off of 12–14 kDa) that had been trimmed to the correct size and secured on both ends with standard clips. The dialysis membrane was put in a receptor compartment containing 50 mL of the studied medium (0.1 M NaOH solution, pH 1.2 or pH 7.4) and swirled at a low rate using a tiny magnetic spin bar. 4 mL of release specimens were withdrawn from the release media and replaced with an equivalent volume of newly heated studied medium (0.1 M NaOH solution, pH 1.2 or pH 7.4) at predefined time intervals (10 min, 15 min, 20 min, 25 min, 30 min, 40 min, 50 min, 1 h, 1.5 h, 2 h, 3 h, 4 h, 5 h, 6 h, 7 h, 8 h, 9 h, 10 h). The absorbance of the drug released was evaluated directly using a blank solution at 302 nm (or 292 nm, 290 nm) *via* (Unicam Helios Alpha UV-vis. spectrometer), and the quantity of ASA released was estimated using the relevant ASA standard calibration curves (Fig. S3†).

#### Drug release kinetics studies

The kinetic release of ASA from loaded LCS microcapsules was determined using zero-order, first-order, and Higuchi kinetic mathematical models (based on the findings from the *in vitro* release investigations). The drug release mechanism was investigated using the Korsmeyer–Peppas model.^56–59^ All the details were mentioned in the ESI.†

## Results and discussion

### Extraction of *Lycopodium clavatum* sporopollenin (LCS) microcapsules and encapsulation of ASA into the extracted LCS microcapsules

In this study, before the ASA encapsulation process, we extracted the internal cytoplasmic materials derived from raw *Lycopodium clavatum* L. spores to create a space and obtain allergen-free outer exines shells (a detailed protocol for sporopollenin extraction from raw spores was mentioned in the experimental section). The shell morphology is determined by the spore or pollen source, and the outer layer's exterior decorating is defined by both the spore or pollen and the empty shell from which it is taken.^29^

**Fig. 1:**
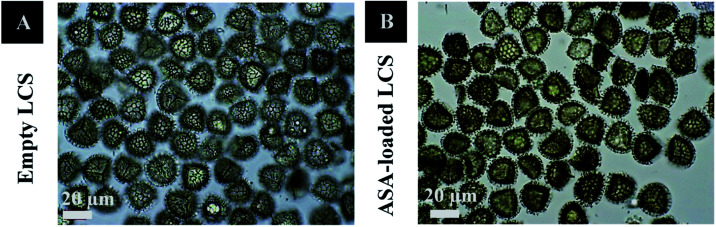
Optical images of (A) hydrated empty LCS and (B) hydrated ASA-loaded LCS.

**Fig. 2:**
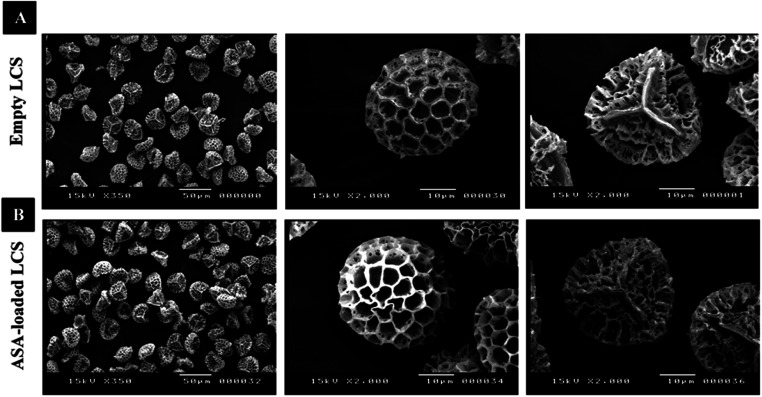
SEM images of LCS microcapsules demonstrating their surface shape; (A) empty LCS prior to ASA loading (B) after ASA loading.

Fig. 1a and 2a, respectively, exhibit optical and scanning electron microscopy pictures of the produced empty LCS microcapsules, illustrating their surface shape and microstructure. They have retained their inherent morphology after the extraction process, indicating the high robustness and resistance of these distinctive sporopollenin biopolymer shells to harsh chemical conditions (treatments), as reported earlier.^29^ This may aid in the drug's resistance to the cruel stomach environment, allowing for oral delivery and enhanced absorption into the gastrointestinal (GI) tract. As illustrated in Fig. 2a, the extracted LCS has an obvious reticular microstructure and ornamentation, as well as trilete scars (Y-shaped), which are distinctive of this spore species.

ASA was encapsulated into the extracted empty LCS microcapsules using a passive-vacuum loading process that done *via* suctioning the LCS suspension in ASA ethanolic solution.

Fig. 1b and 2b depict optical and SEM images of ASA-loaded LCS, demonstrating structural integrity that is critical for encapsulants. Additionally, no accumulation of ASA was noticed on the surface of the ASA-loaded LCS microcapsules, as illustrated by SEM images. The ASA-loaded LCS microcapsules retained their surface morphology without changing in the morphology following the passive-vacuum loading technique, displaying the recognizable ornaments and monodispersity in size of such loaded sporopollenin. Notably, the encapsulated LCS microcapsules were properly rinsed with water/ethanol to eliminate any leftover drug from their surface. The only path for ASA to cross the exine layer and enter the internal cavity of the empty LCS microcapsules is *via* the multidirectional nanochannels in the exine layer that are responsible for the in and out transfer of nutrients to spores. The method by which diverse materials enter or exit the sporopollenin *via* these nanochannels is not fully understood, as the sporopollenin's entire chemical structure is still mostly unknown.^60^ Further, these nanochannels are anticipated to aid in forming the desirable protein-free microcapsules and may be employed to release the active ingredients for drug administration.^29,39^

We have also conducted an elemental analysis to determine whether or not the protein extraction technique from raw spores was successful. The elemental analysis results were gathered in Table S1,† showing that the protein content (*N*% × 6.25) after extraction becomes 0%, indicating the successful extraction of protein from raw spores.

### ASA loading capacity (LC%) and entrapping efficiency (EE%)

UV analysis was used to determine the effectiveness of loading ASA at a 1 : 1 (50 percent) weight ratio (w/w) into empty LCS microcapsules with a theoretical drug loading (TDL) of 50%. Three cycles of probe sonication in 0.1 M NaOH were performed to thoroughly separate the encapsulated ASA from 10 mg of ASA-loaded LCS. The ASA had a loading capacity (LC) of 26.7% and an encapsulation efficiency (EE) of 53.4%, respectively.

Our results suggest that a loading concentration of 0.27 g ASA per 1 g of LCS was achieved successfully, implying a larger loading and consequently a higher EE percentage of ASA compared to earlier experiments utilizing equivalent sporopollenin species loaded with model proteins.^28,61^ The drug loading of 1 : 1 (50%) w/w used in this study was chosen after performing experiments using different wt% ratios of the drug (ASA) and LCS microcapsules. It was found that 1 : 1 w/w loaded microcapsules have more drug loading than others.

**Fig. 3:**
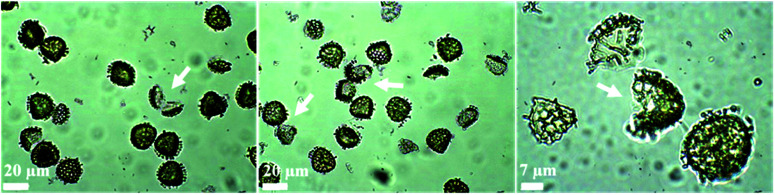
Optical pictures of ultrasonically shattered ASA-loaded LCS microcapsules at various magnifications following the last sonication cycle used to extract ASA for loading capacity tests. The ASA-loaded LCS were subjected to a four-cycle sonication technique, and each cycle consists of two minutes of sonication at 50% amplitude three times. Some sporopollenin ornaments are fragmented (indicated by arrows).

Fig. 3 displays optical images of numerous broken ASA-loaded LCS microcapsules and the subsequent probe ultrasonic extraction of the encapsulated ASA used to measure the loading capacity. Sonication was performed in a four-cycle method, and each cycle was made up of two minutes of sonication at 50% amplitude three times, which was sufficiently powerful to disrupt the sporopollenin architecture and shatter some of them. In our previous study, we demonstrated (using SEM analysis of broken drug-loaded LCS microcapsules after using the same protocol used here) that both the interior core and the LCS surfaces were free of any drug accumulations, indicating the protocol's efficacy in extracting the drug.^62^ Furthermore, our earlier optical microscopic pictures of the drug-loaded LCS following each cycle of the sonication process depicted an increase in sporopollenin ornament fragmentation over the four-cycle process,^62^ corroborating the current findings. Sonication of numerous pollen species culminated in fractured pollens, which aided in obtaining a high-quality FTIR spectrum.^63^

### Fourier-transform infrared (FTIR) spectroscopy analysis

**Fig. 4:**
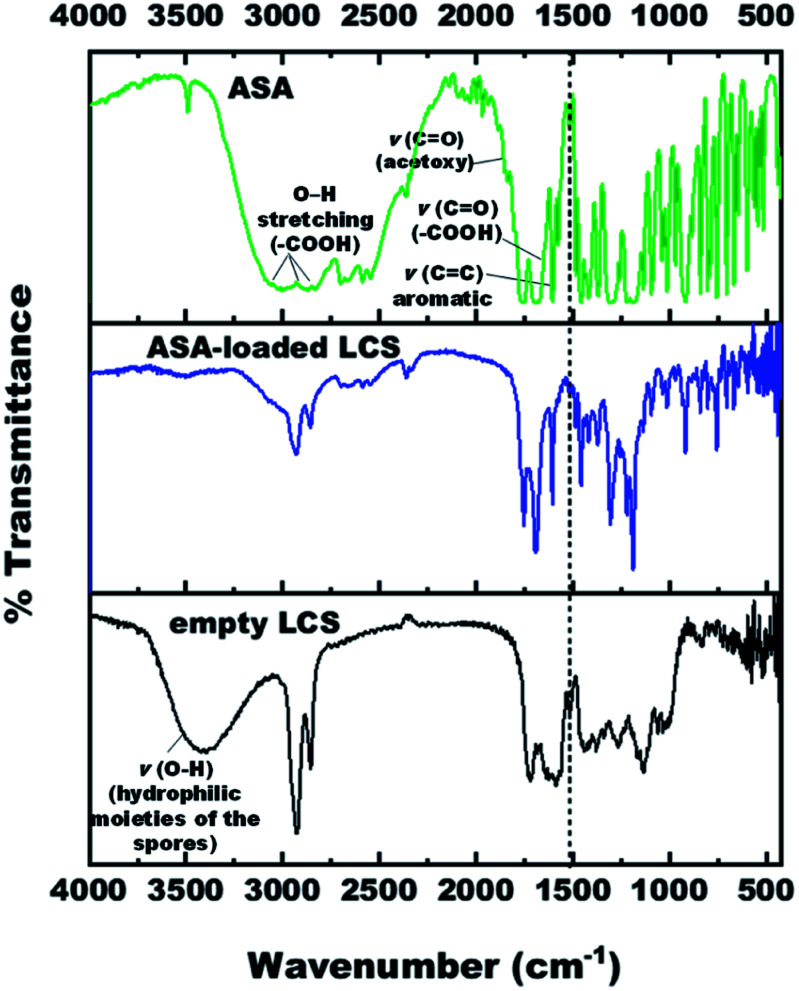
FTIR spectra of empty LCS, pure ASA and ASA-loaded LCS.

The FTIR spectra of empty LCS, pure ASA and ASA-loaded LCS were examined (Fig. 4) to confirm that the ASA is encapsulated within the LCS microcapsules and assess if the ASA interacts with sporopollenin. Table S2† summarises the related principal absorption bands. The FTIR spectrum of empty LCS spores was shown in Fig. S4,† in which a broad vibration *ν*(O–H) band appears at 3398.29 cm^−1^, contributing to the hydrophilic moieties of the spores.^64^ This vibration *ν*(O–H) band is drastically quenched after ASA encapsulation which may be due to the attachment of ASA on hydroxyl and carboxylic group. The band at 1588.37 cm^−1^ is for *ν*_as_(COO^−^), which appears due to the hydrolysis of esters with KOH.^65^ The band at 1717.94 cm^−1^ is characteristic for *ν*(C=O) in alkyl esters or (–COOH). Other characteristic peaks for empty LCS are listed in Table S2.†

The pure ASA showed bands at 3001 cm^−1^, 2871.15 cm^−1^, 2699.21 cm^−1^ are associated with O–H stretching vibration of carboxylic acid groups. The characteristic bands of the ASA FTIR spectrum at 1755.22 cm^−1^, 1702.19 cm^−1^, and 1605.26 cm^−1^ correspond to *ν*(C=O) stretching frequency of ester group (acetoxy group), *ν*(C=O) in (–COOH) carboxylic acid group, and (C=C) skeletal in-plane vibration of the aromatic ring, respectively. Further, the bands at 1308.90 cm^−1^, 1220.41 cm^−1^ and 1094.84 cm^−1^, 1012.94 cm^−1^ are attributed to (C–O) stretching vibration of the carboxylic acid/ester group, respectively (Fig. S5 and Table S2†). The wavenumber assignments of ASA are consistent with the earlier documented literature.^66–69^

For ASA-loaded LCS microcapsules, bands appeared at 1752.56 cm^−1^, 1693.70 cm^−1^, 1605.98 cm^−1^, 1305.55 cm^−1^, 1220.24 cm^−1^, 1094.90 cm^−1^, and 1012.99 cm^−1^, all from ASA, indicating the existence of ASA in the loaded sporopollenin and confirming the encapsulation of ASA successfully into the empty LCS microcapsules. The appearance of absorption bands in the presented spectrum of ASA-loaded LCS microcapsules can be correlated with the characteristic frequencies of functional groups from both components; LCS microcapsules and the ASA. Thus, the absorption bands of the loaded LCS overlap those of the starting components, and they are shown in Fig. S6 and Table S2.†

A unique band at ∼1515 cm^−1^ was noted in the spectra of unfilled and ASA-loaded LCS (Fig. 4 and Table S2†), which was associated with the (C=C) stretching vibration of the sporopollenin's phenolic components.^64^ It is obvious from the spectrum of ASA-loaded LCS (Fig. S6†) that the characteristic bands of sporopollenin and ASA were almost unchanged (appeared without any significant shifts), except changes of intensity of the bands in the spectrum, probably due to incorporation of both spectra together, indicating the absence of chemical interaction between the ASA and the biopolymer sporopollenin microcapsules.

### Thermogravimetric analysis (TGA)

Thermogravimetric analysis was utilized to examine the thermal characteristics of the various specimens used in this experiment in an inert nitrogen environment and determine whether encapsulating ASA within the empty sporopollenin affects thermal stability. Fig. S7† illustrates the TGA of empty LCS, ASA-loaded LCS, and pure ASA up to 600 °C, at which they lose roughly 92.5, 99.6, and 99.1% of their original mass, respectively. The loading percent of ASA was determined by the mass loss difference between ASA-loaded LCS and empty spores at temperatures ranging from 100 to 600 °C (ref. 70) in TGA (Fig. S7†), and the amount was about 20.3%. Together with the SEM and FTIR measurements, this mass gain confirms the success of our ASA encapsulation method.

To obtain a greater understanding of the phases of material impairment across the heating range, first derivative thermal gravimetry (DTG) was used (Fig. S8†). The temperatures at which the major DTG degradation bands exhibit the greatest rate of weight impairment, demonstrated as a percent weight loss, are summarised in Table 1. Mass impairment occurs at zone I as a result of adsorbed water being removed from unfilled LCS and ASA-loaded LCS. For unfilled LCS, the bulk (58.94%) of mass loss noted within zone II (temperature variation 250–500 °C) is due to decomposition of various biomass constituents (hemicellulose, cellulose, and lignin).^71^ However, in zone III (above 500 °C), there is a weight loss which is the residues pyrolysis (*e.g.*, spores wall and sporopollenin).

**Table 1:** The temperature of maximum weight impairment rate from DTG for empty LCS, ASA, and ASA-loaded LCS

Sample	Maximum temperature (°C)/weight loss (Wt.L%)		
	*T*_max1_/Wt.L	*T*_max2_/Wt.L	*T*_max3_/Wt.L
Empty LCS	45.1	275.7 (58.9%)	440.8 (13.7%)
ASA	138.9 (38.0%)	293.2 (60.4%)	—
ASA-loaded LCS	115.6 (22%)	291.08 (39.4%)	421.6 (35%)

The DTG and TGA curves of pure ASA demonstrated two distinct phases of decomposition (mass losses in two consecutive steps) between about 120 and 350 °C (Fig. S7 and S8† and Table 1). It is described as a complex decomposition process of acetylsalicylic acid. In zone I, the first breakdown phase was shown at 138.9 °C temperature, equating to a 38.0% weight loss. In zone II, the second decomposition stage happened at a temperature of 293.2 °C with a maximum rate of weight loss of 60.4%.

**Scheme 2:**
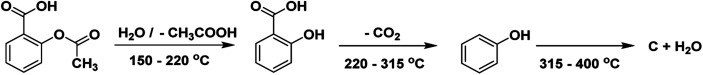
The thermal decomposition of the acetylsalicylic acid.

It has been proposed (performing FTIR analysis of ASA *vs.* temperature during the TGA stability research) that the first (complex) process eliminates the acetic acid (characteristic odor) and forms the salicylic acid. The second (complex) process involves removing CO_2_ and forming the phenol. The elimination of CO_2_ begins in the first process. Finally, the phenol is decomposed, and the carbonated residuum suffers a complete burning, together with a slight endothermic effect. Chemical reactions corresponding to the described processes are presented in Scheme 2,^72^ as reported in previous studies.^72,73^ It was previously demonstrated that salicylic acid, with a melting point of 159 °C, evaporates immediately following the first decomposition stage. This is why the first mass-loss step passes directly into the following step.^73^ It was suggested that along with the evaporation of salicylic acid, a decomposition process also occurs, resulting in the formation of phenol and CO_2_ (ref. 73) (Scheme 2).

Moreover, with ASA-loaded LCS microcapsules, a fairly robust peak developed inside zone I in DTG (Fig. S8†), centered at 115.6 °C, equivalent to a 22% weight loss, which was not seen in empty LCS before loading, indicating ASA encapsulation. Additionally, we found that the last peak of empty LCS in zone III, attributed to residue pyrolysis, has vanished from the ASA-loaded LCS DTG curve (Fig. S8† and Table 1). The DTG outcomes gathered for ASA-loaded LCS indicated that integration of ASA into the LCS microcapsules did not significantly affect ASA's thermal stability.

### X-ray diffraction (XRD) analysis

**Fig. 5:**
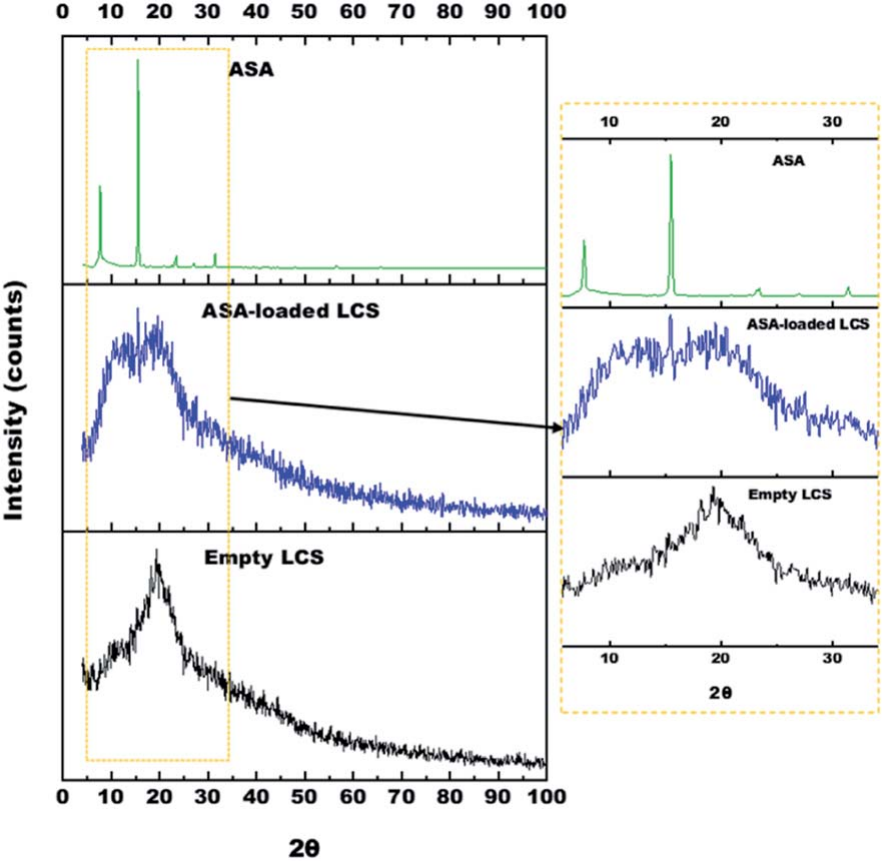
XRD pattern for pure ASA (the sharp and well-defined peaks indicate the crystalline nature of the ASA), ASA-loaded LCS and empty LCS. The XRD pattern on the right is a zoom-in of the 2*θ* at 15–35° region of all samples, allowing for a better observation of the characteristic peaks.

X-ray diffraction (XRD) was used to measure the encapsulation's efficacy by checking the crystallinity of the ASA placed into the unfilled LCS microcapsules. Fig. 5 shows the XRD Pattern for empty LCS, ASA-loaded LCS, and pure ASA. Pure ASA's XRD pattern shows major diffraction peaks at 7.7, 15.5, 22.5, 23.4, 27, 31.4, and 32.6°, confirming the expected crystallinity structure of the pure ASA. These findings agree with other reports.^66,74,75^ The empty LCS microcapsules exhibited a characteristic amorphous form with a single prominent peak at 2*θ* = 19.3°.^76^

The ASA-loaded LCS microcapsules revealed three characteristic peaks at 8, 15.5 and 19.5°. The last peak (19.5°) revealed to LCS microcapsules, however, the other two peaks at 8, 15.5° revealed to amorphous and crystalline ASA, respectively. Since not all loaded ASA was encapsulated into LCS, therefore, some ASA was encapsulated (53.4% based on EE%), however, the other free ASA remains on the LCS surface. During the encapsulation process the drug may appeared in the amorphous form rather than the crystalline form.^77^ Consequently, the encapsulated ASA appears to be present in the amorphous form inside the LCS rather than crystalline form which clearly appears in the peak at ∼8°. However, the free ASA on the surface remains in the crystalline form which gives the peak (with lower intensity compared to Pure ASA's XRD) at ∼15°.

### Ultraviolet-visible (UV-vis) spectroscopy

**Fig. 6:**
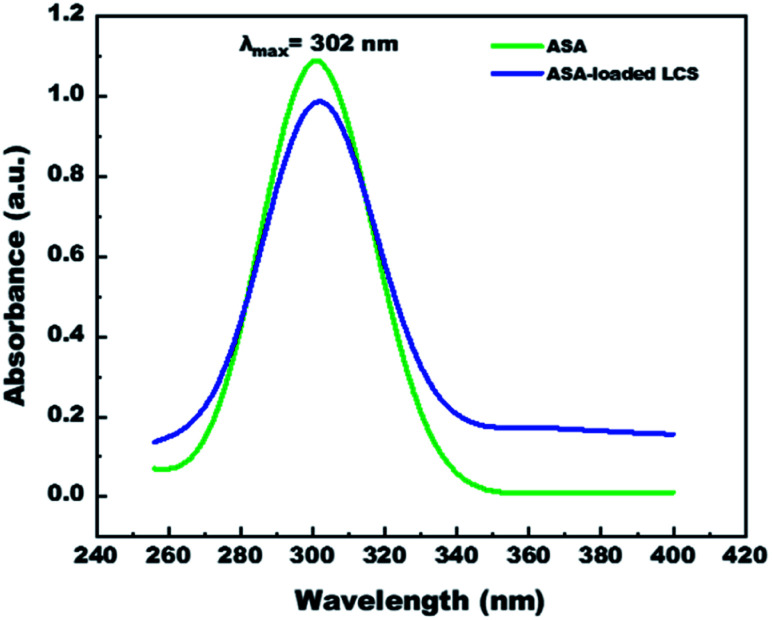
UV-visible spectra of pure ASA and ASA-loaded LCS, in 0.1 M NaOH solution.

We have measured UV-visible spectroscopy for further confirming the successful loading and/or interaction of the ASA into the cavities of the empty LCS microcapsules after the encapsulation process. Fig. 6 demonstrates the UV-visible spectra of pure ASA and ASA-loaded LCS scanned from 200–400 nm in 0.1 M NaOH solution. As shown in Fig. 6, the characteristic absorption peak position of pure ASA at *λ*_max_ = 302 nm does not chemically shift after loading compared with the ASA-loaded LCS spectrum, indicating that the loading process has been successfully done without interaction between LCS and ASA. Additional analyses, including SEM-FTIR, validated these findings.

### *In vitro* ASA release from ASA-loaded microcapsules by using dialysis membrane

**Fig. 7:**
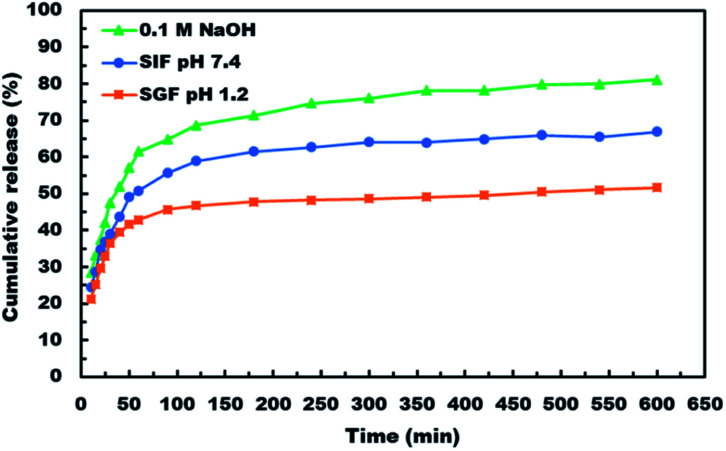
*In vitro* release profile of ASA from ASA-loaded LCS microcapsules in various pH media.

After demonstrating the microencapsulation of ASA in LCS microcapsules, we examined the *in vitro* release profile of ASA from ASA-loaded LCS microcapsules to investigate the potential of these encapsulation platforms for delivering ASA in a sustained and controlled way. In this regard, we conducted *in vitro* release experiments of ASA from ASA-loaded LCS under SGF and SIF circumstances that resemble those found in the GI tract, as illustrated in Fig. 7. Apart from these activated GI tract settings, ASA was also released in 0.1 M NaOH (pH ∼ 13) for comparison purposes. As shown in Fig. 7, the cumulative ASA release percentages in SGF, SIF, and 0.1 M NaOH were 21.3%, 24.5%, and 28.4%, respectively, with a slight burst effect during the first ten minutes. Additionally, we determined that the overall ASA release from ASA-loaded LCS microcapsules was 51.7, 66.9, and 81.1% after 10 hours in SGF, SIF, and 0.1 M NaOH, consecutively. The results depicted in Fig. 7 suggested a slow release of ASA from ASA-loaded LCS under acidic SGF circumstances. In contrast, a quicker release profile was observed in both SIF (pH 7.4) and 0.1 M NaOH (pH ∼ 13), which can be related to ASA's variable solubility in various mediums.

In all cases, the *in vitro* release profiles of ASA were pH-dependent and revealed a total percentage release in biphasic profile starting with an initial small burst release followed by a plateau within 1 h, with 42.9, 50.8 and 61.6% of total ASA drug released in SGF, SIF and 0.1 M NaOH, respectively in the first 1 h, accompanied by a sustained release profile for the next 9 h. The initial burst effect could result from the ASA dissolving rapidly in the receptor medium for the ASA adsorbed on the surface of the ASA-laden LCS microcapsules or for the ASA accessible near the inside surface of the loaded LCS microcapsules. In contrast, the sustained profile could be due to ASA molecules imprisoned deep within the LCS microcapsules' cores. Further, the receptor media may require time to penetrate, dissolve, and release the ASA medicine encapsulated within the LCS microcapsules *via* the nanochannels on their surfaces.

ASA is a weak acid with pH-dependent solubility, based on its solubility and behavior under various situations. The solubility of ASA rises with rising pH above its acid dissociation constant (p*K*_a_) value of 3.5 or 3.49, depending on the study goal.^78^ The Henderson–Hasselbalch equation states that a weak acid's solubility grows exponentially with pH until it reaches the solubility of the ionized form.^78^ It was established that a medium with a pH value less than the p*K*_a_ value for weak acids slows weak acid dissociation, resulting in a higher concentration of the non-ionized form.^3^ This result is consistent with those obtained at pH 1.2, which shows a lower cumulative release percentage due to ASA's lower solubility in this pH value than other studied pH conditions. Owing to the growing surface area for absorption and porous membranes, ASA is more absorbed in the small intestine.^3^ In this respect, our slow ASA release in SGF, together with the controlled release of the LCS carrier, may be seen as a good strategy for minimizing the frequency of SGF dosing to avoid stomach irritation, extending ASA release at the favored absorption site, and ultimately increasing its bioavailability.

ASA would dissolve much faster in intestinal fluids than in gastric fluids because of their higher pH, supporting the results obtained from release at pH 1.2 (SGF) and pH 7.4 (SIF). Higher pH and better absorption would result in faster dissolution and removal of potentially irritating drug particles from the intestine. The release of ASA in 0.1 M NaOH has a higher cumulative percentage of release because ASA ionizes and hence has a significantly greater solubility in basic solutions and may convert ASA to its sodium salt, which is based on an acid–base reaction. Although acetylsalicylic acid is often administered orally as a free acid, different salts are also available, including aluminium, calcium, and sodium salts. Sodium acetylsalicylate or calcium acetylsalicylate are the usual forms of soluble aspirin sold.^78^

Thus, our findings indicated that the pH-dependent release profile of ASA-loaded LCS may indicate a quicker release rate in the intestines, thereby enhancing the desired sustained release. Compared to other reported studies using the same type of sporopollenin, the *in vitro* release profiles illustrated in Fig. 7 indicated a significantly slower and more regulated release,^46^ or sunflower pollens.^79^ The findings of *in vitro* tests indicate that the LCS microcapsules are suited for drug delivery systems that provide sustained and regulated release under a variety of pH values observed in the gastrointestinal tract. Pharmacists and engineers can design regulated medication delivery systems that preserve the therapeutic agent effect by controlling the rate of drug release. In comparison to other reported ASA release encapsulants, the sustained release properties of our ASA-loaded LCS microcapsules may be a strong competitive encapsulation innovation.^2,80^ Nonetheless, the sporopollenin isolated from *Lycopodium clavatum* had some advantages over the other encapsulants, including superior chemical stability, size uniformity, mucoadhesion, and the capacity to penetrate the gut wall and reach the bloodstream.^29,35^

### Release kinetics at different pH circumstances

Typically, relevant mathematical models are assigned to understand the *in vitro* drug release mechanisms better. The interpretation of the release process can be helped by fitting several kinetic models to the experimental data.^58,59^ First, zero- and Higuchi models were applied to our *in vitro* ASA release data (Fig. S9†), and the Korsmeyer–Peppas model was also utilized. The resulting characteristics are shown in Table 2.

**Table 2:** Kinetic models for ASA release from ASA-loaded LCS at different pH conditions^a^

Kinetic model	Zero-order		First order		Higuchi		Korsmeyer–Peppas		
	*R* ^2^	*k*_0_	*R* ^2^	*k*_1_	*R* ^2^	*K*_H_	*R* ^2^	*k*_kp_	*n*
pH 1.2 (SGF)	0.573	0.035	0.625	0.0007	0.724	1.086	0.826	18.230	0.176
pH 7.4 (SIF)	0.655	0.057	0.731	0.0012	0.809	1.728	0.957	12.583	0.327
0.1 M NaOH	0.689	0.073	0.819	0.0021	0.835	2.202	0.995	10.134	0.443

a *R* ^2^: correlation coefficient, *k*: release rate constant, *n*: release exponent.

The correlation coefficient with the highest degree of correlation (*R*^2^) identifies the most acceptable mathematical model of drug release kinetics.^57^ As demonstrated in Table 2, the Higuchi model had a greater *R*^2^ value for ASA release in SGF, SIF, and 0.1 M NaOH mediums, revealing that the release kinetics followed a diffusion process.^59^ After establishing that the primary mechanism of ASA release is diffusion-controlled in all media, the kind of diffusion mechanism must be assigned using the Korsmeyer–Peppas semi-mathematical model. The Korsmeyer–Peppas formula is only significant to the first 60% of the release profile (*M*_*t*_/*M*_∞_ less than 0.60), where *M*_*t*_ and *M*_∞_ signify the mass of medication released at a certain time and at equilibrium, consecutively. The “*n*” in the Korsmeyer–Peppas formula is used to analyse the one-dimensional mechanism of drug release for different non-swellable devices.^81^ According to Korsmeyer–Peppas model, when the diffusional release exponent (*n*) is ≤0.5, Fickian diffusion is considered, whereas if 0.5 < *n* < 1.0, anomalous non-Fickian transport is ascribed.^58^ Under this regard, we discovered that *n* less than 0.5 for ASA release in acidic SGF, somewhat basic SIF, and strongly basic 0.1 M NaOH conditions, showing that ASA is released *via* the Fickian diffusion transport pathway from ASA-loaded LCS sporopollenin. Consequently, the results indicated that ASA was released from ASA-loaded LCS microcapsules *via* a time-dependent process governed by Fickian diffusion. In conclusion, the release profile of ASA from ASA-loaded LCS microcapsules exhibited two unique phases: a burst release of poorly encapsulated ASA on the surface is followed by a phase of sustained-release rates controlled by diffusion.

## Conclusions

We have explored the feasibility of microencapsulation of ASA within sporopollenin microcapsules obtained from natural *Lycopodium clavatum* (LCS) spores to provide controlled and sustained delivery platform. LCS sporopollenin microcapsules were successfully extracted from their raw spores. ASA was encased within an empty LCS using a mixture of passive-vacuum and (1 : 1) w/w loading techniques. According to UV data, ASA had a loading capacity (LC) of 26.7% and an encapsulation efficiency (EE) of 53.4%. The FTIR, SEM, TGA-DTG, and XRD analyses demonstrate that ASA was successfully loaded into the LCS. The absence of residual ASA on the surface of the ASA-loaded LCS microcapsules indicates effective encapsulation, as confirmed by SEM analysis. The FTIR analysis revealed no interaction between the ASA and the empty LCS. The *in vitro* release of ASA from the ASA-loaded LCS was studied under various pH values that mimicked those found in the GI tract. We found that SGF had a slow-release rate, whereas SIF had a rapid release rate, indicating that the release was pH-dependent. The release of ASA was attributed to a Fickian diffusion process in SGF, SIF, and 0.1 M NaOH, as determined by fitting several mathematical models to experimental *in vitro* release data. Our findings indicate that these resilient LCS microcapsules could be useful for ASA's regulated and sustained delivery, creating a new generation of innovative oral and topical medication delivery capabilities. This controlled carrier could reduce the side effects of ASA, which are caused by its increased release into the GI tract.

## Conflicts of interest

The authors have no conflicts of interest to disclose.

## Author contributions

Al-Shymaa Mohammed: investigation, formal analysis, data curation, writing – original draft. Amro Dyab: conceptualization, methodology, writing – original draft, supervision, writing – review and editing. Fouad Taha: conceptualization, supervision, methodology, writing – review and editing. Ahmed Abd El-Mageed: writing – reviewing and editing, supervision, methodology.

## Supplementary Material

RA-012-D2RA02888C-s001
