# Supplementary material for: Pollen-derived microcapsules for aspirin microencapsulation: in vitro release and physico-chemical studies
Source: RSC Adv. 2022 Aug 10;12(34):22139–49. doi: 10.1039/d2ra02888c (PMC9364082; doi:10.1039/d2ra02888c)
Supplement: RA-012-D2RA02888C-s001 [file RA-012-D2RA02888C-s001.pdf]

Electronic Supplementary Material (ESI) for RSC Advances.
This journal is © The Royal Society of Chemistry 2022

ESI

Pollen-Derived Microcapsules for Aspirin Microencapsulation: *In Vitro* Release and Physico-Chemical Studies

Al-Shymaa Y. Mohammed[a], Amro K.F. Dyab[a], Fouad Taha[a] and Ahmed I. A. Abd El-Mageed[b,a]*

[a] *Colloids & Advanced Materials Group, Chemistry Department, Faculty of Science, Minia University, Minia 61519, Egypt.*

[b] *Chemistry Department, Faculty of Science, GALALA University, Galala City, Suez 43711, Egypt.*

**Corresponding Author* (Ahmed I. A. Abd El-Mageed) *E-mail: ahmed.abdelmageed@mu.edu.eg*

Methods

ASA-loading capacity and encapsulation efficiency

10 mg of ASA-loaded LCS was suspended in 5 mL of 0.1M NaOH, stirred for 15 minutes and sonicated using an ultrasonic probe device (Ivymen® ultrasonic homogenizer, CY-500, Spain) at room temperature for 2 minutes in three cycles at 50% amplitude. After the sonication process, the suspension was filtered and 1 mL of the filtrate was diluted up to 10 mL then heated to 37 °C. In order to determine the quantity of the ASA encapsulated into the microcapsules, the absorbance of the clear heated solution (diluted filtrate) was measured by UV-vis spectrophotometer (Unicam Helios Alpha) at 302 nm using fresh 0.1M NaOH solution as a blank. ASA content was determined using the relevant calibration curve, taking the dilution factor into consideration. These steps were repeated four times to extract almost all drug in the loaded LCS, by extract the loaded LCS already used in the first time from filter paper and suspended it again in 5 mL of fresh 0.1M NaOH, then stirred, and use the ultrasonic under the same conditions mentioned above. However, in the second and third cycles, the filtrate was not diluted before UV analysis since the ASA concentration was sequentially decreased. The following equations were used to evaluate the loading capacity (LC%) and the encapsulation efficiency (EE%):

Loading capacity (%) = (amount of drug entrapped) / weight of drug loaded sporopollenin) ×100

Encapsulation efficiency (%) = (practical drug loading / theoretical drug loading) ×100

Preparation of phosphate buffer saline (PBS, pH = 7.4)

Phosphate buffer saline was prepared by adding 8 g of sodium chloride, 0.2 g of potassium chloride, 1.44 g of di-sodium hydrogen orthophosphate and 0.24 g of potassium di-hydrogen orthophosphate to 800 mL distilled water, then mixed them well and adjusted the final volume to 1L with distilled water. If necessary, pH can be adjusted using HCl or NaOH.

Drug release kinetics studies

Zero-order, first-order and Higuchi kinetic mathematical models were used to determine the release kinetics of ASA from the loaded LCS microcapsules using the data obtained from the *in vitro* release studies. The Korsmeyer-Peppas model was used to find out the mechanism of drug release. [1-4] The zero-order kinetics model can be expressed the following equation:

$$Q_t = Q_0 + K_0 t$$

Where Q_t is the cumulative amount of drug released at time t, Q_0 is the initial amount of drug in the matrix, K_0 is the zero-order rate constant and t is the time. In order to explain the release kinetics of ASA, cumulative amount of drug released is plotted versus the time. The first order kinetics model can be expressed by the equation:

$$\log C = \log C_0 - K_1 t/2.303$$

Where C is the percent of drug remaining at time t, C_0 is the initial amount of drug in the solution, K_1 is the first-order rate constant and t is the time. Log cumulative % of drug remaining is plotted versus time, and -$K_1/2.303$ is the slope of the obtained line. Higuchi model based on Fickian diffusion mechanism is expressed by the simplified Higuchi equation:

$$Q_t = K_H \times t^{1/2}$$

Where K_H is the Higuchi constant and t is the time. To determine the drug release kinetics, cumulative amount of the released drug is plotted against the square root of time. Korsmeyer-Peppas model to understand the dissolution mechanisms from the matrix is expressed by the equation:

$$Mt/M\infty = K_{kp}t^n$$

$Mt/M\infty$ is a fraction of drug released at time t,

$$\log (Mt/M\infty) = \log K_{kp} + n \log t,$$

Mt is the amount of drug released in time t, $M\infty$ is the amount of drug released after time ∞, n is the diffusional exponent or drug release exponent, K_{kp} is the Korsmeyer release rate constant. To study release kinetics a graph is plotted between log cumulative % drug release log $(Mt/M\infty)$ *vs*. (log t), n value is used to characterize different release mechanisms.[3]

Scanning electron microscopy (SEM)

Scanning electron microscope analysis was acquired using JSM-5400 LV JEOL (Japan). Samples were coated with 20 nm gold using a gold sputter (JEOL JFC-1100E). An acceleration voltage of 15 kV was used during capturing the images at different magnifications to observe the surface morphology.

UV-vis spectrophotometry

The UV-vis spectrophotometric analyses were performed with a double beam Unicam Helios Alpha spectrophotometer (made in England) fitted with deuterium and tungsten lamps, using a scan range of 200 - 400 nm. Quartz cuvettes with optical path length of 1 cm were used.

pH-measurements

The pH was measured using a pH-meter model HI 8014 from HANNA instruments (Portugal) with Adwa (AD 1230B) gel-filled pH electrode. The pH-meter was calibrated before use with standard buffer solutions of pH= 4, 7 and 10 at 25 °C.

Optical microscopy

Optical images were taken by an OPTIKA (B-293) microscope (Italy) fitted with Optikam B5 (model; 4083.B5) digital camera and the images were processed using Optika IS view software. Micrometer slide

was used for software calibration by using the microscope at different magnifications to calculate scale bars of images in microns. Calibration was made at the same resolution and magnification used through imaging.

Combustion CHN elemental analysis

CHN elemental analysis was conducted using a calibrated VarioEL III CHN automatic elemental analyzer (Elementar, Germany). Prior to conducting elemental analysis, to ensure combustion efficiency, all samples were dried at 60 °C for a minimum of 1 h before being combusted in excess oxygen at high temperature to release compositional carbon %C, hydrogen %H and nitrogen %N. The percentage of protein in different samples was calculated using the obtained percentage of nitrogen and the total Kjeldahl Nitrogen (TKN) conversion factor of 6.25 in accordance to recommendations from the Association of Analytical Communities (AOAC) to transform weight percent of nitrogen into weight percent of protein using the equation: [5]

$$\% \text{ Protein} = \% \text{ Nitrogen} \times 6.25$$

Fourier-transform infrared (FTIR) spectroscopy analysis

FTIR spectra were obtained using a FTIR instrument (Thermo Scientific Nicolet iS10 spectrometer, USA). Samples were ground with anhydrous potassium bromide (Spectrosol grade) with a ratio of 1/9 (w/w) to obtain disks. All spectra were obtained in the 400 - 4000 cm^{-1} range as a result of scan against a background, employing OMNIC software.

X-ray Diffraction (XRD) and Thermogravimetric Analysis (TGA)analysis

XRD was measured using a JEOL JSX-60PA diffractometer equipped with Ni-filtered Cu Kα radiation, at a wavelength (λ) of 1.5418Å, 35 kV voltage, and a current of 30 mA. The diffraction data were recorded in the range of 4°-100° with 0.1° 2θ step size.

TGA analysis was carried out with a TGA-50H from (SHIMADZU- Japan), with a heating rate of 10 °C/min up to 600 °C in a flow of nitrogen at 20 mL/min.

Other instruments and data processing software

Other lab instruments were used i.e., (Ivymen ultrasonic homogenizer, model CY-500, Spain), thermostated water bath (Fisher Scientific Isotemp Refrigerated bath Circulator, model 9000), mechanical stirrer-heater device model Ingenieurbüro CAT, M. Zipperer GmbH, type: M 6.1, made in Germany. Origin 2018 64 Bit software was used for statistical treatment of the data and the construction of graphs. Derivation of the spectra was performed with Vision V3.40 UV-vis spectrophotometry software.

Supplementary Figures

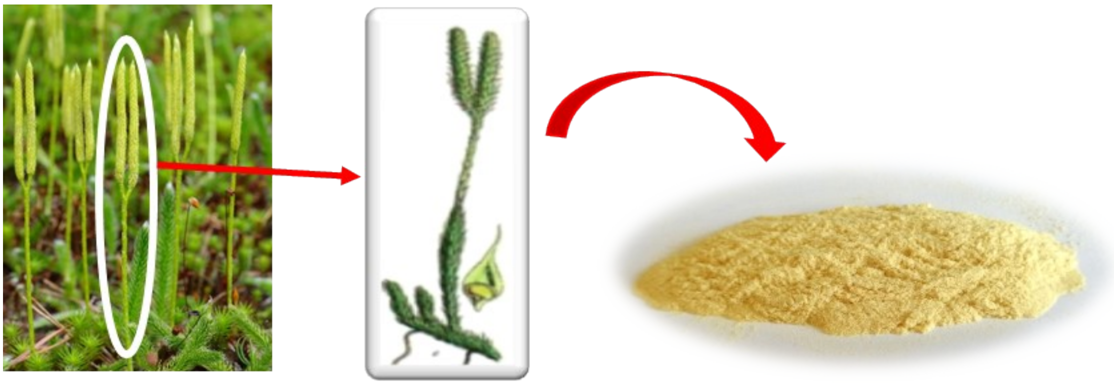

Figure S1. Digital images of raw *Lycopodium clavatum* L. spore powder and its plant.

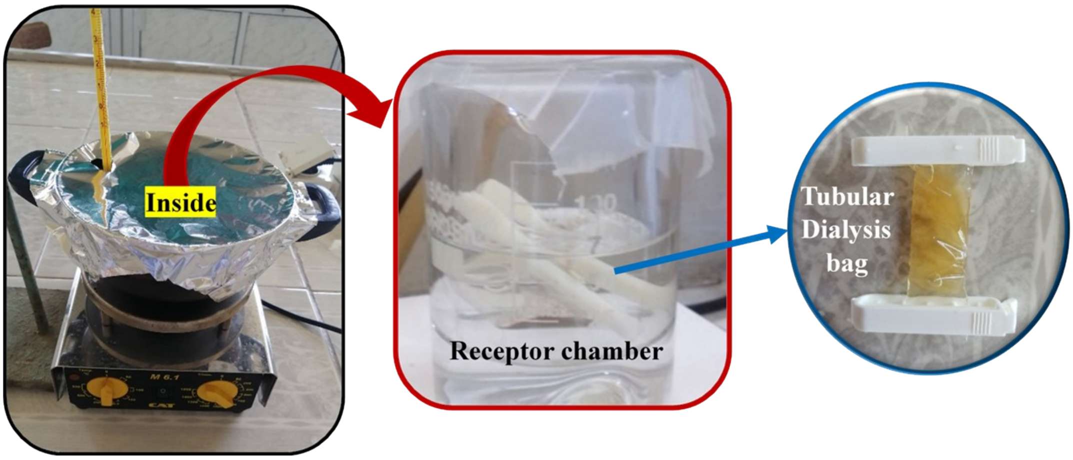

Figure S2. *In vitro* release set up.

Calibration curves of ASA at the three media with different pHs

Preparation of standard stock solutions, determination of maximum wavelengths (λ_{max}) and preparation of calibration curves of ASA in different media were done as follows. ASA was used without further purification. All experiments were performed at 37 °C. The UV spectrum and calibration curve of ASA solutions were obtained at three media with different pHs i.e. 0.1M NaOH (pH ~ 13), 0.1M HCl (pH = 1.2 ± 0.05) and a phosphate buffer saline (PBS) (pH = 7.4 ± 0.02). Standard stock solutions of ASA (0.1 mg/mL) for obtaining calibration curves for 0.1M NaOH, pH = 7.4 and pH = 1.2 were prepared by dissolving the same quantity of ASA (5 mg) in the same quantity (50 mL) of three media 0.1M NaOH, pH = 7.4 and pH = 1.2. The stock solutions were scanned via a UV-vis. spectrophotometer at 37 °C between 200-400 nm for determining the maximum wavelengths (λ_{max}) for each solution. To prepare the calibration curves; these stock solutions were suitably diluted to obtain many different calibration standards within the range of 0.01 - 0.1 mg/mL (10 - 100 µg/mL) in 0.1M NaOH, pH = 1.2, and in the range of 0.02 - 0.09 mg/mL (20 - 90 µg/mL) in pH = 7.4. For each given medium, the maximum wavelength absorption of ASA was used i.e. 290 nm for acidic medium (pH = 1.2) samples, 302 nm for basic medium (0.1M NaOH) samples and 292 nm for phosphate buffer (pH = 7.4) samples. The UV-vis. spectra with (λ_{max}) and calibration curves of ASA at the three media with different pHs, are shown in Figure S3.

1) In 0.1M NaOH (pH ~ 13), (10 to 100 μg/ml)

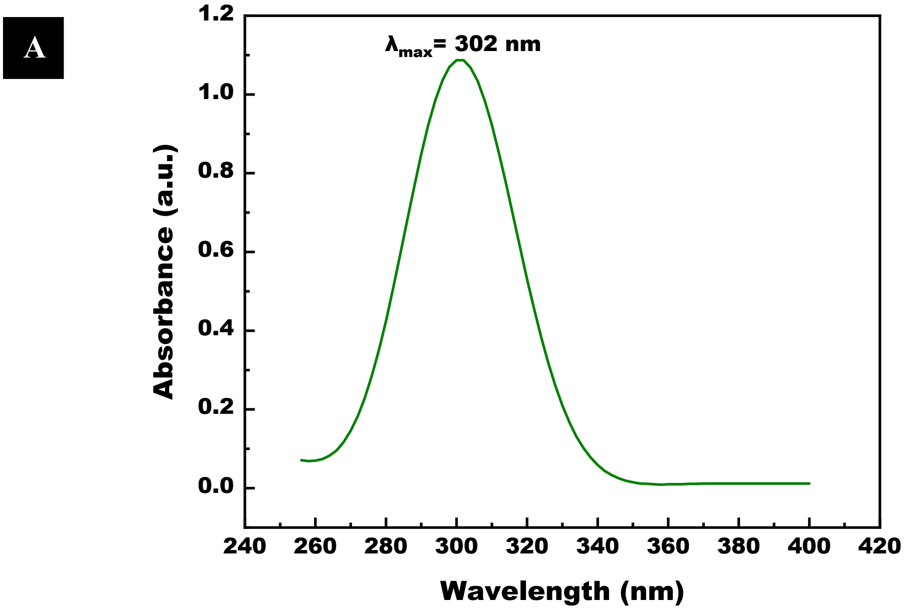

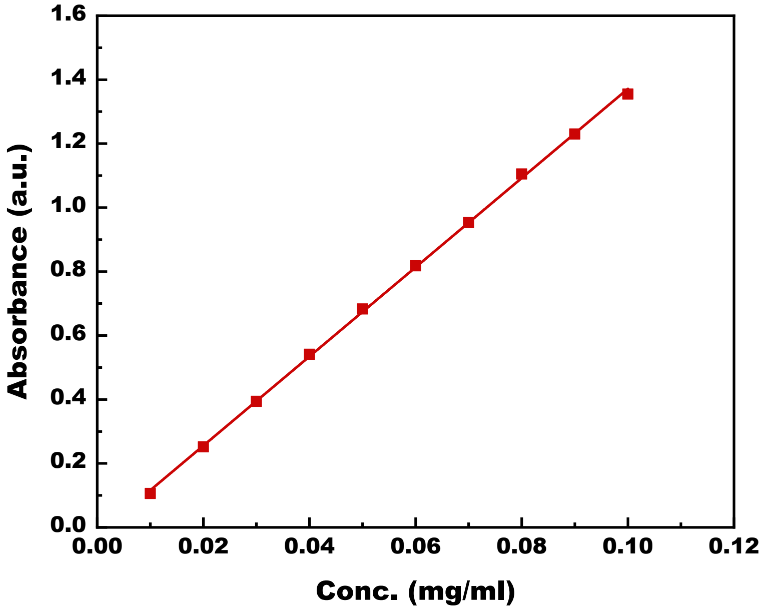

Equation	y = a + b × x
Intercept	- 0.0234
Slope	13.94727
R-Square (R^2)	0.99957

2) In pH=7.4 ± 0.02 (Phosphate buffer saline (PBS)

B

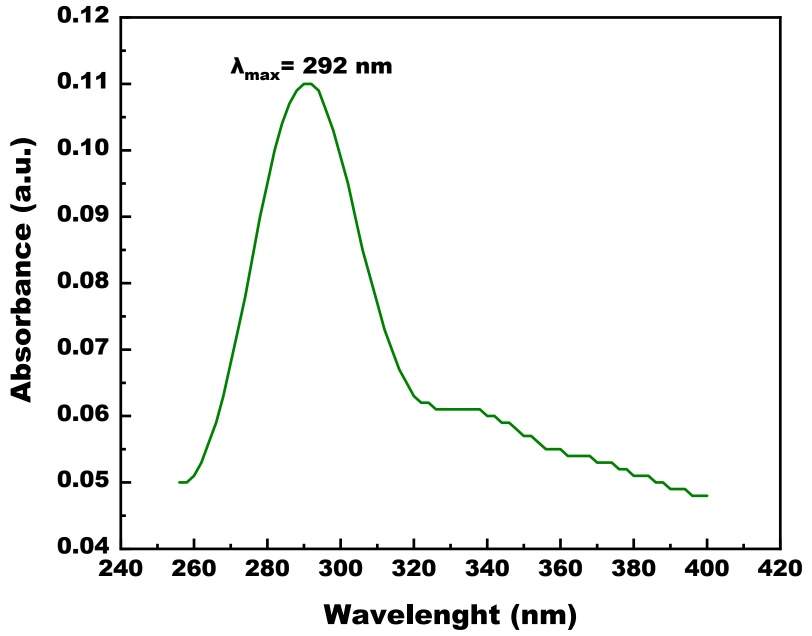

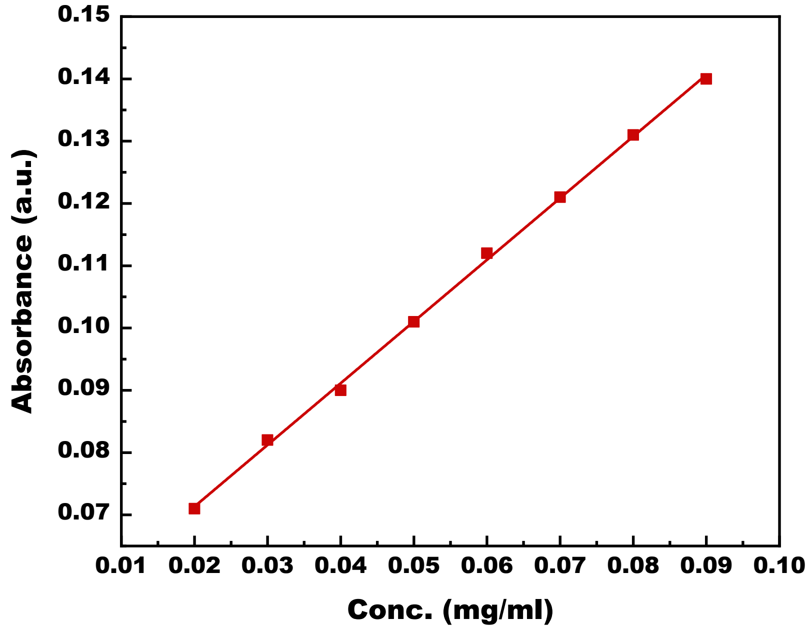

Equation	y = a + b × x
Intercept	0.05152
Slope	0.99048
R-Square (R^2)	0.99912

3) In pH=1.2 ± 0.05 (0.1M HCl)

C

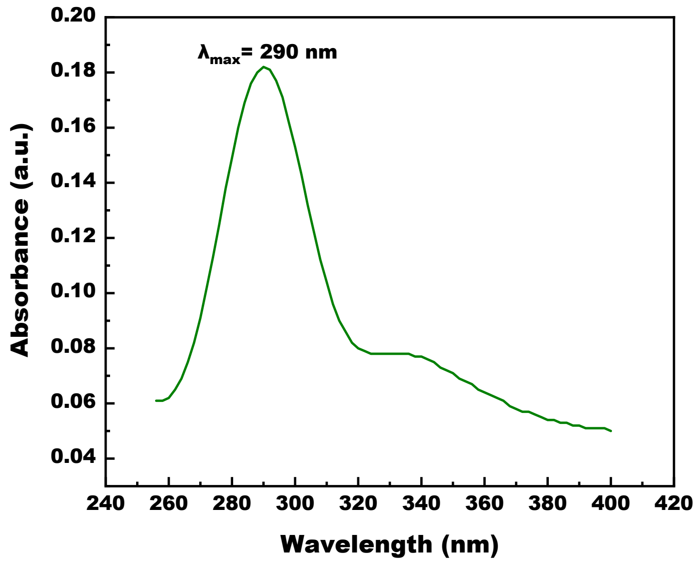

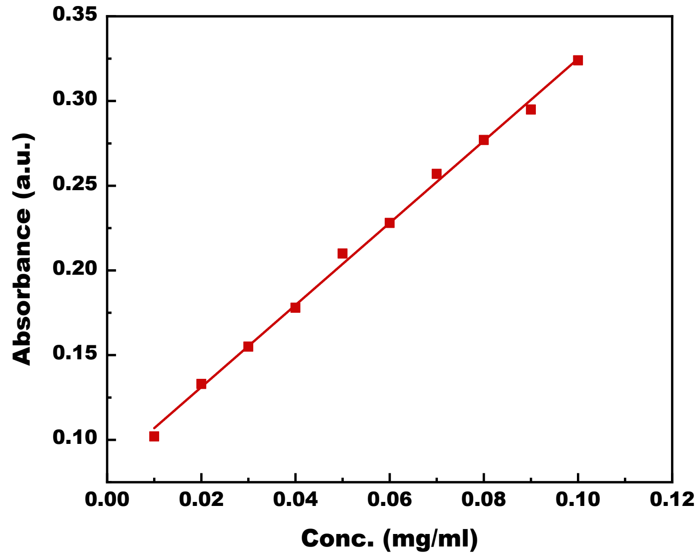

Equation	y = a + b × x
Intercept	0.08267
Slope	2.42242
R-Square (R^2)	0.99743

Figure S3. UV-visible spectra and calibration curves (from 10 to 100 µg/mL or from 20 to 90 µg/mL) of ASA at three media with different pH values at 37 ˚C: In **(A)** 0.1M NaOH (pH ~ 13), **(B)** pH = 7.4 (PBS) and **(C)** pH = 1.2 (0.1M HCl).

FTIR Spectra

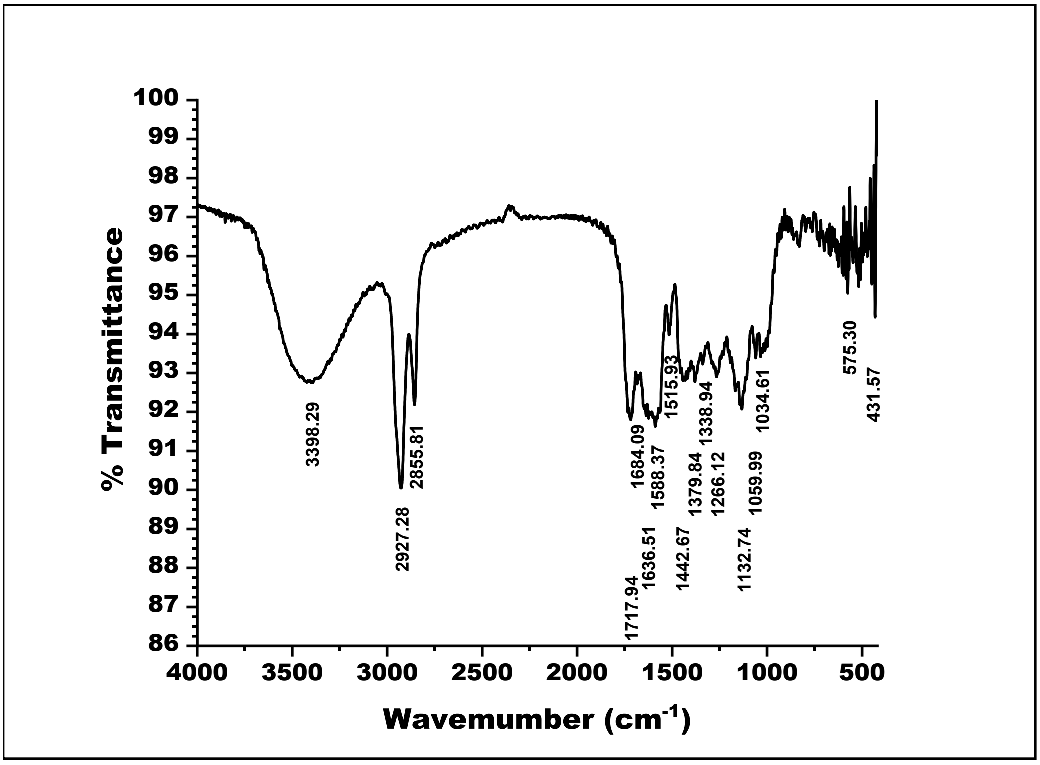

Figure S4. FTIR spectrum of empty LCS.

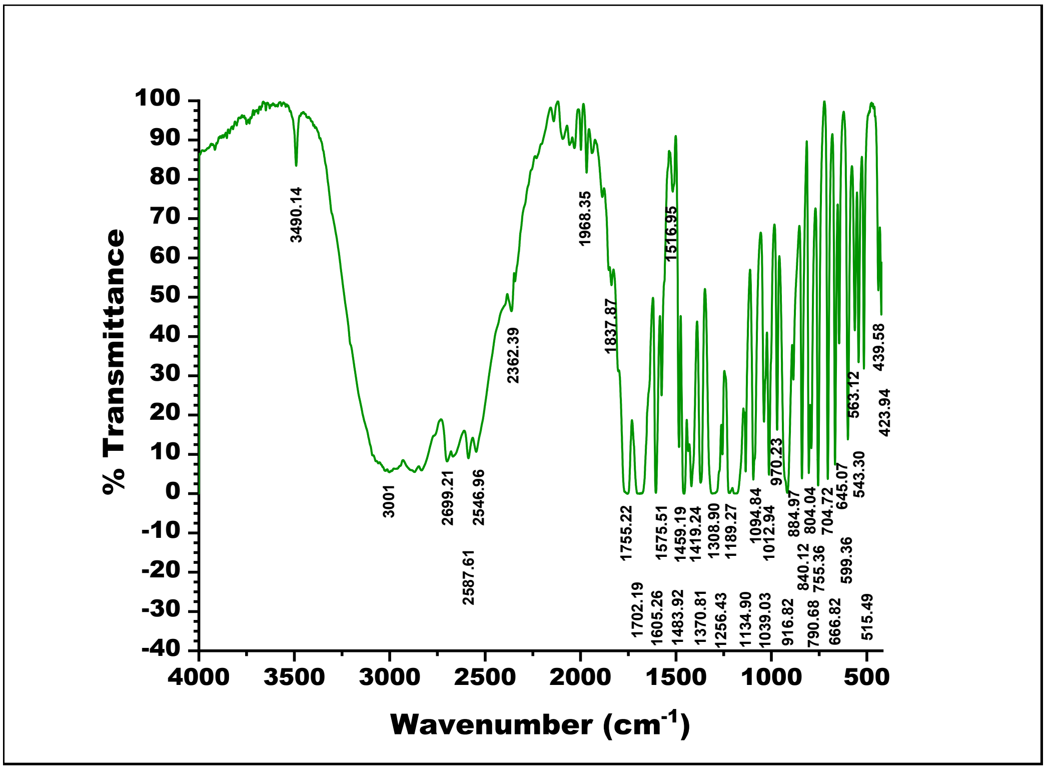

Figure S5. FTIR spectrum of pure ASA.

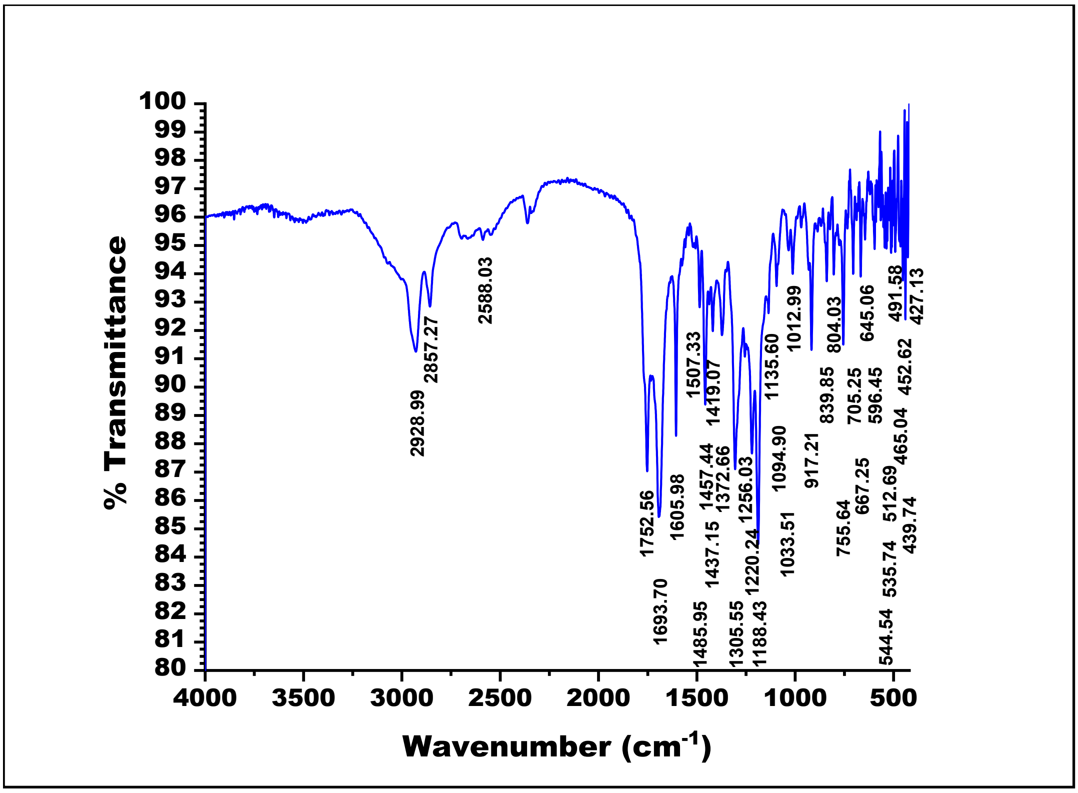

Figure S6. FTIR spectrum of ASA-loaded LCS.

Thermogravimetric analysis (TGA)

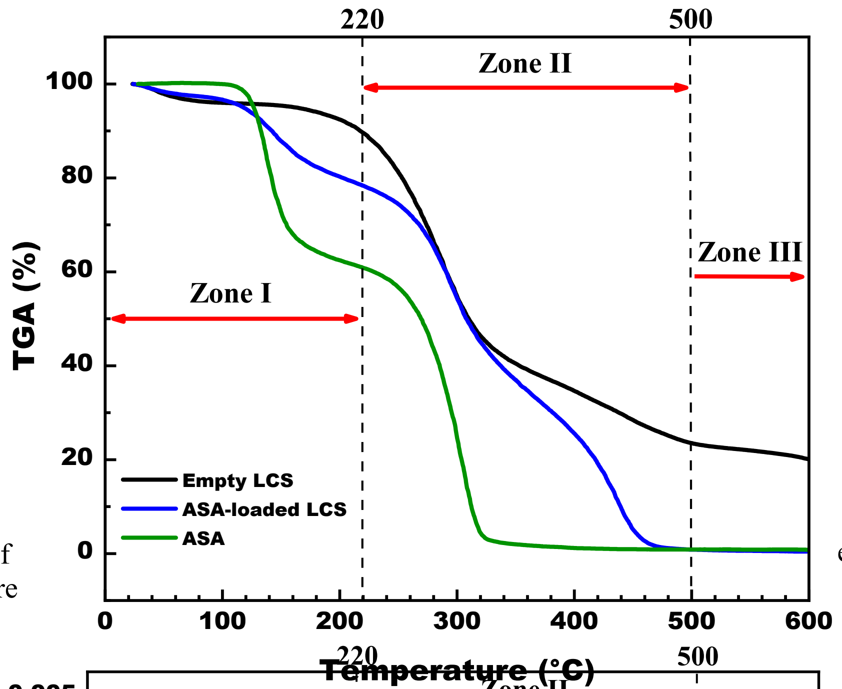

Figure S7. TGA of empty LCS, ASA-loaded LCS and pure ASA.

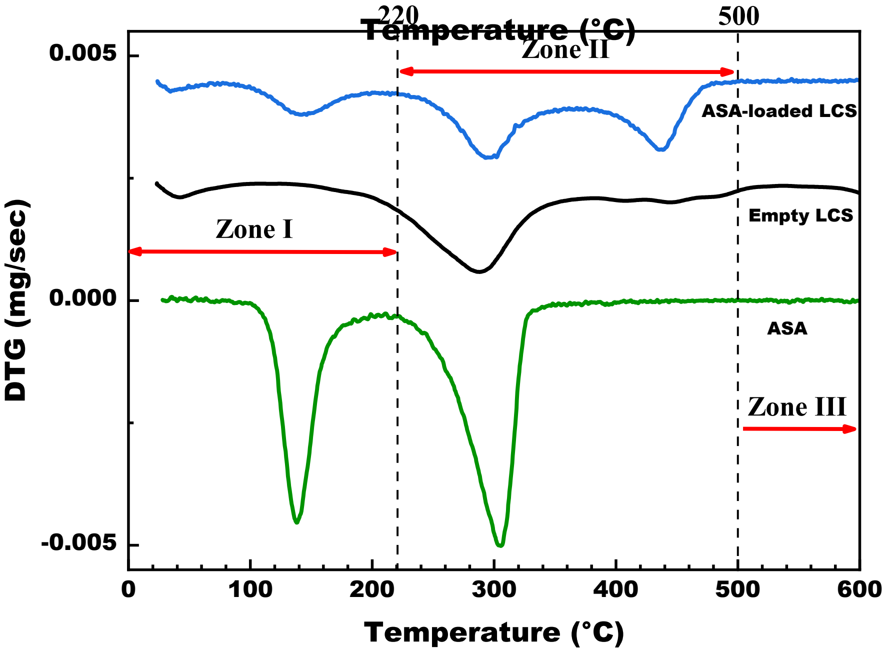

Figure S8. DTG of ASA-loaded LCS, empty LCS and pure ASA.

Release kinetics at different pH conditions

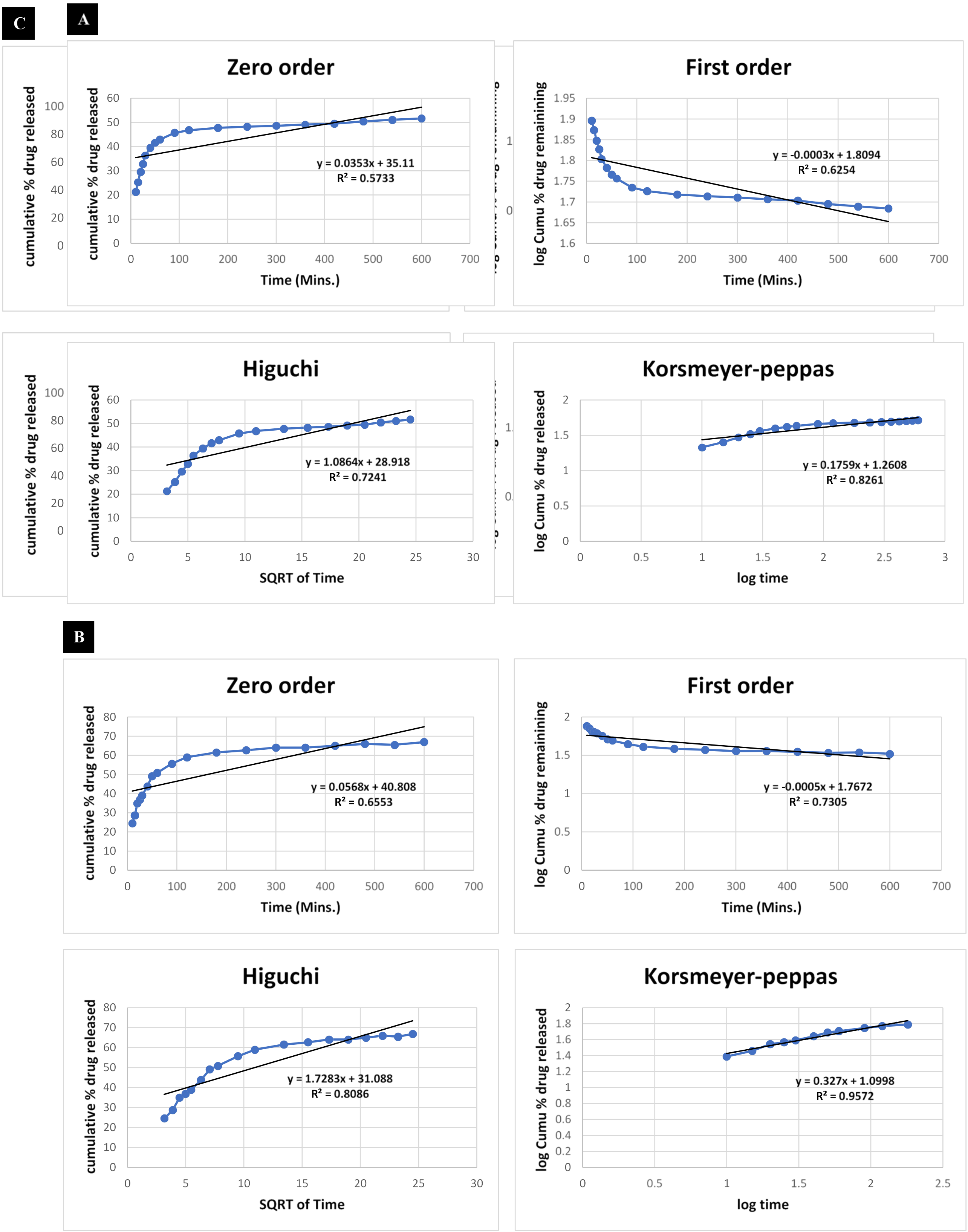

Figure S9. Kinetic models for ASA release from ASA-loaded LCS at different pH conditions: In **(A)** pH = 1.2 (SGF), **(B)** pH = 7.4 (SIF) and **(C)** 0.1M NaOH (pH ~ 13).

Supplementary Tables

Table S1. Elemental analysis (wt.%) of raw spores and treated LCS sporopollenin

Sample name	Carbon	Hydrogen	Nitrogen
Raw spores	67.09	9.44	1.25
Treated LCS	55.08	7.28	0.00

Table S2. Major FTIR absorption bands of empty LCS sporopollenin, ASA-loaded LCS and pure ASA.

Sample	FTIR band (cm^{-1})	Assignment[a]
Empty LCS	3398.29	ν(O-H) in carboxylic acids, alcohol, phenol or carbohydrates
	2927.28, 2855.81	ν_{as}(C-H) and ν_s(C-H) of (-CH_2)
	1717.94	ν(C=O) in alkyl esters or (-COOH)
	1636.51	ν(C=C)
	1588.37	ν_{as} (COO^-) in carboxylate anions [5]
	1515.93	ν(C=C) in phenolic components, sporopollenin [6, 7]
	1442.67	δ(C-H) in aromatic, sporopollenin [6]
	1379.84	δ(C-H) in methyl (-CH_3) of lipids or carbohydrates
	1338.94	C-C and C-O skeletal vibrations in cellulose
	1266.12	ν(C-O) stretching in carbohydrates
	1132.74	ν_{as} (C-O-C) in cellulose [5]
	1059.99	ν(C-O-C) in cellulose [8]
	1034.61	ν(C-O-C) pyranose ring vibration [8]
ASA-loaded LCS	3503.21	ν(O-H)
	2928.99, 2857.27	ν_{as}(C-H) and ν_s(C-H) of (-CH_2)
	1752.56	ν(C=O) of ester group of ASA
	1693.70	ν(C=O) in (-COOH) carboxylic acid group of ASA
	1605.98	ν(C=C) skeletal in-plane vibration of aromatic ring of ASA
	1507.33	ν(C=C) in phenolic components, sporopollenin [6, 7]
	1437.15	δ(C-H) in aromatic, sporopollenin [6]
	1419.07	β (O-H)
	1372.66	δ(C-H) in methyl (-CH_3) of lipids or carbohydrates
	1305.55, 1220.24	ν(C–O) acid
	1135.60	ν_{as} (C-O-C) in cellulose [5]
	1094.90, 1012.99	ν(C–O) ester; β (C–H)
	1033.51	ν(C-O-C) pyranose ring vibration [8]
	839.85	δ(C-H) aromatic ring in sporopollenin [9]; γ (C–H) of ASA
ASA	3490.14	ν(O-H)
	3001, 2871.15, 2699.21	ν(O-H) of carboxylic acid group
	1755.22	ν(C=O) of ester group
	1702.19	ν(C=O) in (-COOH) carboxylic acid group
	1605.26	ν(C=C) skeletal in-plane vibration of aromatic ring
	1575.51	ν(C–C)
	1516.95	ν(C=C) aromatic ring
	1483.92	δ_{as} (-CH_3)
	1419.24	β (O-H)
	1370.81	δ_s (-CH_3)
	1308.90, 1220.41	ν(C–O) acid
	1189.27	β (C–H)
	1094.84, 1012.94	ν(C–O) ester; β (C–H)
	1039.03	β (C–H); Ring breathing
	916.82	γ (O-H)
	840.12, 790.68, 755.36, 704.72	γ (C–H)
	563.12, 543.30	ρ (CO_2)

[a] ν = stretching vibration, ν_s = symmetric stretching vibration; ν_{as} = asymmetric stretching vibration; δ = bending vibration; δ_s = symmetric bending; δ_{as} = asymmetric bending; γ = out–of–plane bending; β = in–plane bending; ρ = rocking.

References:

1. D. Wojcik-Pastuszka, J. Krzak, B. Macikowski, R. Berkowski, B. Osinski and W. Musial, *Materials*, 2019, **12**, 1202.
2. H. Baishya, *J. Dev. Drugs*, 2017, **06**.
3. R. W. Korsmeyer, R. Gurny, E. Doelker, P. Buri and N. A. Peppas, *Int. J. Pharm.*, 1983, **15**, 25-35.
4. J. Siepmann and N. A. Peppas, *Int. J. Pharm.*, 2011, **418**, 6-12.
5. M. J. Uddin, S. Liyanage, N. Abidi and H. S. Gill, *J. Pharm. Sci.*, 2018, **107**, 3047-3059.
6. P. E. Jardine, F. A. J. Abernethy, B. H. Lomax, W. D. Gosling and W. T. Fraser, *Rev. Palaeobot. Palynol.*, 2017, **238**, 1-6.
7. M. J. Uddin, N. Abidi, J. Warzywoda and H. S. Gill, *ACS Appl. Mater. Interfaces*, 2019, **11**, 20628-20641.
8. M. El-Sakhawy, S. Kamel, A. Salama and H.-A. S. Tohamy, *Cellul. Chem. Technol.*, 2018, **52**, 193-200.
9. M. Bağcıoğlu, B. Zimmermann and A. Kohler, *PLoS One*, 2015, **10**, e0137899.
